# Feasibility and priority strategies for diabetes prevention and control in Chinese demonstration areas

**DOI:** 10.1080/16549716.2025.2543605

**Published:** 2025-08-20

**Authors:** Jing Li, Jing Yang, Jiayu Feng, Xiaohui Xu, Tingling Xu, Maigeng Zhou, Wenlan Dong, Yanbo Zhang

**Affiliations:** a Department of Health Statistics, School of Public Health, Shanxi Medical University, Jinzhong, China; b Shanxi Provincial Key Laboratory of Major Diseases Risk Assessment, Shanxi Medical University, Jinzhong, China; c National Center for Chronic and Noncommunicable Disease Control and Prevention, Chinese Center for Disease Control and Prevention, Beijing, China; d Department for Chronic and Non-communicable Disease Control and Prevention, Shandong Center for Disease Control and Prevention, Shandong, China

**Keywords:** Importance-Performance Analysis, chronic disease prevention, health policy evaluation, priority setting, implementation science

## Abstract

**Background:**

Diabetes is a leading global public health challenge with 537 million cases worldwide in 2021 and significant economic and societal burdens. Despite global initiatives, feasibility constraints limit widespread implementation of diabetes prevention/control measures. The key to facilitating the implementation of diabetes prevention and control measures lies in prioritise them.

**Objectives:**

This study aimed to determine the priority of diabetes prevention and control measures in China from the feasibility perspective.

**Methods:**

From January to February 2021, an online survey was administered to professionals specifically responsible for implementing chronic disease prevention and control programs in 488 national demonstration areas in China. The survey assessed the importance and feasibility of 44 diabetes prevention and control measures across 10 dimensions. The Importance-Performance Analysis (IPA) model categorized these indicators into four quadrants: Highest Priority (high importance and feasibility), Priority Improvement (high importance, low feasibility), Lowest Priority (low importance and feasibility), and Secondary Improvement (low importance, high feasibility).

**Results:**

Quadrant 1 (‘Highest Priority’) included health education, community action, high-risk discovery and intervention, and medical insurance and family doctors, which should be maintained and strengthened. Quadrant 2 (‘Priority Improvement’) included patient management and complication screening, necessitating targeted efforts to enhance implementation. Quadrant 3 (‘Lowest Priority’) included personal health services assessment follow-up, environmental support, sugar reduction policy, and diabetes co-infection prevention, indicating a risk of overinvestment. No indicators were categorized into Quadrant 4 (‘Secondary Priority’).

**Conclusions:**

Prioritizing diabetes prevention and control measures based on feasibility maximizes the utilization of limited resources, providing implementable recommendations for policymakers.

## Paper Context

- **Main finding**: The study identified the priority of diabetes prevention and control measures in China’s demonstration areas based on feasibility and importance, using the Importance-Performance Analysis. Key high-priority areas included health education, community action, high-risk discovery and intervention, and medical insurance services, which were deemed both important and feasible for implementation.
- **Added knowledge**: This study is the first to assess the priority of diabetes prevention and control measures from a feasibility perspective, offering a practical framework for allocating limited resources in large-scale public health initiatives.
- **Global health impact for policy and action**: This study highlights the critical importance of prioritizing diabetes prevention and control measures based on both their importance and feasibility. The findings offer valuable guidance for global health policy, encouraging governments to focus on practical, high-impact interventions that are both actionable and sustainable, ensuring effective use of resources in addressing the global diabetes epidemic.

## Background

Diabetes has emerged as a prominent global public health challenge that exerts a substantial impact on the overall well-being of individuals. The Global Diabetes Atlas provides compelling evidence of a persistent escalation in diabetes prevalence, with a continuous year-on-year increase [1]. In fact, 2021 witnessed a staggering worldwide tally of 537 million diabetes cases, representing a notable increase of 74 million cases compared with the data reported in 2019 [2]. In addition to its significant health burden, diabetes also imposes a substantial economic cost in China. A recent study estimated that diabetes-related productivity losses in China reached approximately USD 2.6 trillion in 2017 [3].

Furthermore, diabetes holds a paramount position as a leading cause of global mortality, accounting for 12.2% of all deaths in 2021. Notably, China has emerged as the country with the highest number of diabetes patients, positioning it as one of the most severe and prevalent chronic diseases in the country [3], thereby exacerbating economic and societal burdens [4]. The risks associated with diabetes complications further strain the overburdened healthcare system [5], as individuals diagnosed at an advanced stage may require intensified healthcare services [6]. Timely diabetes prevention and control interventions are critically important for averting or postponing complications, preventing premature mortality, and enhancing the overall quality of life.

The World Health Organization initiated the Global Diabetes Compact in 2021, to mitigate the risk of diabetes and ensure equitable access to comprehensive, affordable, and high-quality treatment and care for all individuals with diabetes [7]. In response to the growing burden of chronic diseases, China launched the National Integrated Demonstration Area for the Prevention and Control of Non-communicable Diseases (NIDAN) in 2010. These demonstration areas, designated at the county or district level through national selection, integrate prevention, early detection, and management of chronic diseases within existing public health systems. Selection criteria included leadership commitment, healthcare capacity, coordination ability, and prior public health experience. Each site receives technical and policy support while developing locally adapted strategies. As of 2020, 488 demonstration areas had been established across China, covering diverse regions and over 250 million people. The NIDAN program serves as a cornerstone of China’s chronic disease control strategy, emphasizing government leadership, multi-sectoral collaboration, and comprehensive interventions. Further details on the program’s selection and implementation framework have been published previously [8].

Despite global efforts to prevent and control diabetes, many cost-effective and evidence-based interventions have not been widely implemented in practice [9]. For example, in Iran, the national diabetes prevention and control program identified weak policy formulation and planning as major contributors to implementation challenges, including economic constraints, workforce shortages, and limited technical capacity [10]. These real-world barriers highlight the critical need to not only identify effective interventions but also systematically evaluate their feasibility before large-scale adoption. In public health systems, particularly in low- and middle-income countries, resource limitations, healthcare workforce shortages, cultural adaptation issues, high implementation costs, and insufficient technical infrastructure often hinder the effective scaling-up of interventions [11,12]. Therefore, prioritizing measures that are both important and feasible allows policymakers to better allocate limited resources, improve scalability, and maximize long-term public health impact, especially within large-scale, resource-constrained healthcare systems.

Current research primarily evaluates the effectiveness of diabetes prevention and control measures, but most studies have focused on individual interventions without considering their feasibility within broader policy frameworks [13–15]. Notably, there is a lack of research on how to prioritize diabetes prevention and control strategies, especially in large-scale systems such as China’s demonstration zones. This study seeks to address this gap by assessing and prioritizing diabetes prevention and control measures within these zones.

To define the priority of current diabetes-focused strategies, this study focused on China’s demonstration areas. Authentic evaluations were collected from key personnel responsible for implementing chronic disease prevention and control measures, assessing both the feasibility and importance of diabetes prevention and control interventions. A progressive analytical framework was employed: first, an Importance-Performance Analysis (IPA) model categorized indicators into initial priority quadrants; second, a second-order confirmatory factor analysis was conducted to determine the relative priority of dimension-level indicators based on standardized second-order factor loadings, while simultaneously ranking the priority of individual measures within each dimension based on first-order factor loadings; third, a partial least squares structural equation model (PLS-SEM) was applied to explore the structural relationships among these dimensions; finally, a hierarchical sunburst visualization was constructed to integrate the quadrant classifications, dimension priorities, and specific control measures. This multi-stage approach provides a comprehensive and evidence-based basis for guiding effective and context-appropriate diabetes prevention and control strategies.

## Methods

### Study population

This study was conducted in China’s National Demonstration Areas for Chronic Disease Prevention and Control. All 488 demonstration areas officially designated under the National Integrated Demonstration Area for the Prevention and Control of Non-communicable Diseases (NIDAN) program by the Chinese Ministry of Health were included. These demonstration areas were selected nationwide through a standardized evaluation process, based on criteria such as government leadership, healthcare infrastructure, multi-sectoral coordination, and experience in chronic disease prevention [8]. As of 2020, 488 demonstration areas had been established in 31 provincial-level administrative divisions. These areas cover 17.1% (488/2846) of counties, cities, and districts nationwide, encompassing more than 250 million people. Specifically, there were 184 areas in the eastern region, accounting for a coverage rate of 27.2%, 152 areas in the central region, with a coverage rate of 14.0%, and 152 areas in the western region, with a coverage rate of 14.0%.

### Participant

The survey participants were local public health professionals directly responsible for implementing chronic disease prevention programs in each demonstration area. These respondents provided context-specific evaluations based on their practical implementation experience, taking into account local economic resources, healthcare capacity, cultural norms, policy environments, and operational challenges. Such practice-based assessments ensured that the collected data accurately reflected the practical feasibility and importance of each intervention across diverse local settings. The NIDAN Working Office issued formal invitations through official government channels to each demonstration area, requesting them to designate qualified professionals with relevant practical experience to complete the survey. Each demonstration area was provided with individualized login credentials to access the online survey system.

### Data collection

Data were collected via a structured, self-administered questionnaire entitled ‘Database Survey of Diabetes Prevention and Control Measures.’ The questionnaire was developed based on a comprehensive literature review of chronic disease prevention and control, including both diabetes-specific studies and broader public health policy documents. The final instrument covered 44 specific intervention measures organized under 10 dimensions: four dimensions for individual-level interventions (high-risk discovery and intervention, patient management, complication screening, diabetes co-infection prevention) and six for population-level interventions (community action, sugar reduction policy, environmental support, health education, personal health services assessment follow-up, medical insurance with family doctor services). Each dimension contained 2 to 8 detailed items. The full questionnaire is provided in Supplementary Table S1.

The survey was conducted online between January and February 2021 via the Demonstration Areas Dynamic Management Information System (https://www.ncdshifanqu.cn/index), an electronic platform developed and maintained by the NIDAN Working Office. Detailed written instructions were provided to all participants, and technical support was available throughout the data collection period.

Each demonstration area submitted one completed questionnaire via the assigned online account. The responses were reviewed by trained auditors to ensure accuracy, completeness, and consistency. In total, 469 valid questionnaires were received, yielding a response rate of 96.11%.

### Definitions and scoring of relevant indicators

Importance: The importance of the intervention measures was assessed by considering their effectiveness, and population coverage (particularly among low-income groups). These factors were integrated to determine the significance of each measure.

Feasibility: Feasibility is comprehensively evaluated from the following four aspects:

1. Accessibility: Refers to how easily the intervention is available to the target population. The greater the ease with which the target population can obtain the intervention, the higher the level of accessibility;
2. Technical simplicity: Assesses the level of expertise and knowledge required to implement the intervention. A lower level of expertise and knowledge needed makes the measure easier to implement and increases its simplicity;
3. Economic feasibility: Total amount of resources and costs required to implement intervention measures. Higher feasibility is associated with lower resources and cost requirements.
4. Cultural acceptability: Examines the consistency between intervention measures and local political forms, ethnic beliefs, and other cultural awareness. A higher level of cultural acceptability indicates that the measures are more readily accepted by the local society and population.

All indicators were evaluated using a 5-point Likert scale, ranging from 1 (lowest) to 5 (highest). The composite feasibility score was calculated as the unweighted mean of these four sub-scores (accessibility, technical simplicity, economic feasibility, cultural acceptability), rounded to the nearest whole integer. The Cronbach’s alpha coefficients for the importance and feasibility of the questionnaire are 0.969 and 0.976, respectively, indicating excellent consistency and reliability of the questionnaire.

### Quality control

To ensure data quality, a multi-level quality control process was implemented. All respondents were provided with standardized instructions and were encouraged to consult with their local teams when completing the survey to reflect collective expert opinion. Completed questionnaires underwent initial verification by trained staff in each demonstration area, followed by centralized auditing by the NIDAN Working Office. Discrepancies or missing data were clarified directly with the respondents before finalizing the dataset.

### Statistical analysis

Continuous variables are presented as $\bar{x}\pm s$. Data description and statistical analyses were conducted using R 4.2 software, with a significance level of α = 0.05.

Division by quadrants: The IPA was employed to divide the data into quadrants. IPA is a widely used analytical tool in market research and service quality management [16]. By visualizing user feedback data, the model helps managers identify which factors require improvement, which factors are performing well, and which factors are less important or need minimal attention, thereby providing valuable insights to support strategic decision-making. The feasibility and importance scores of each dimension indicator were used as the evaluation parameters. The mean values of these indicators served as reference lines for dividing the matrix into four quadrants. The evaluation indicators were allocated to a two-dimensional coordinate graph:

- Quadrant 1, (Highest Priority): Measures located in this quadrant are of high importance and high feasibility, which should be maintained and strengthened.
- Quadrant 2, (Priority Improvement): Measures in this quadrant are of high importance but low feasibility, and require prioritization for improvement.
- Quadrant 3, (Lowest Priority): Measures here are of both low importance and low feasibility, indicating a risk of overinvestment; resource allocation should be optimized.
- Quadrant 4, (Secondary Improvement): Measures in this quadrant are of low importance but high feasibility, suggesting that resources may be allocated for improvement.

Indicator ranking: Control measures were treated as manifest variables, dimensional indicators as first-order latent variables, and individual- and community-level as second-order latent variables. Second-order confirmatory factor analysis was conducted to determine the relative importance of the dimensional indicators using standardised factor loading coefficients. The dimensional indicators within the same quadrant as determined by the IPA model were ranked. Building on the importance – feasibility IPA model, a quadrant bubble chart was constructed using the magnitudes of the second-order standardised factor loading coefficients as the bubble sizes. Bubble sizes visually represented the relative importance of each dimension, with larger standardized factor loadings corresponding to larger bubbles. The control measures indicators were ranked based on standardised first-order factor loadings to visualise the sorting of dimension indicators and control measures within each quadrant using a sunburst plot.

Path analysis: Based on the prioritised ranking of the dimension indicators, a Partial Least Squares Structural Equation Model (PLS-SEM) was used to examine the interrelationships among the ten dimension indicators. PLS-SEM is suitable for exploratory research involving complex latent structures and small to moderate sample sizes, with relatively relaxed assumptions about data distribution [17,18]. Path coefficients were employed to explore the associations between the dimension indicators.

## Results

**Figure 1.**
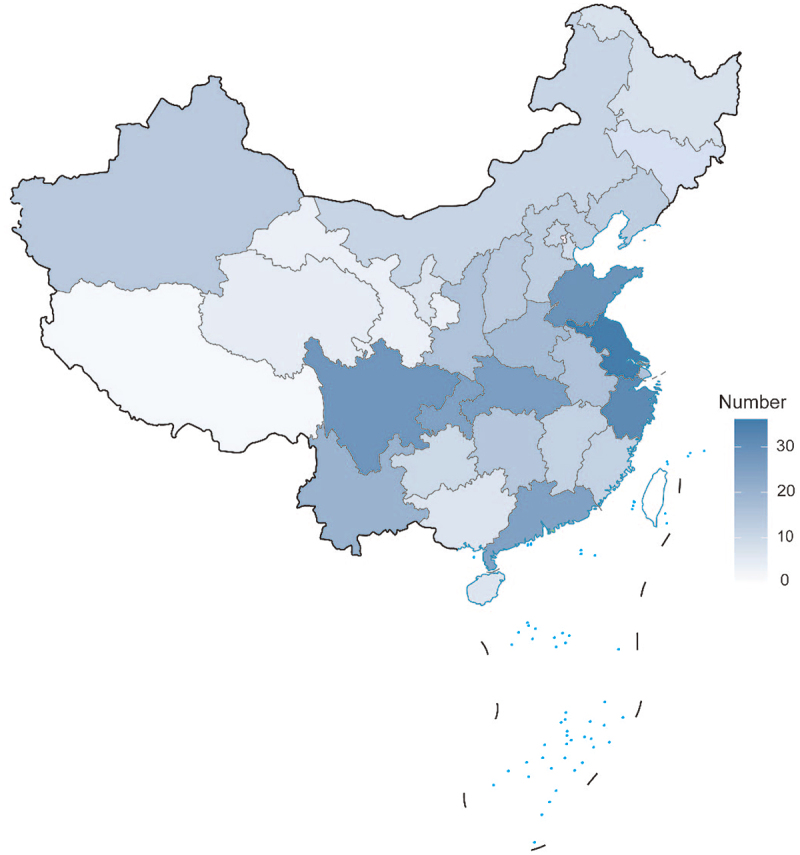
Distribution of valid completed questionnaires in demonstration areas in different provinces of China (examination drawing number: GS (2019)1698). Jiangsu Province contributed the highest number of valid responses with a total of 36, while Tibet Autonomous region had the lowest number of valid responses with only2.

This survey encompassed all 488 demonstration areas in China, yielding 469 valid responses, resulting in a response rate of 96.11%. Figure 1 presents the geographic distribution of the 469 valid responses obtained from the national demonstration areas.

### IPA model quadrant division

**Figure 2.**
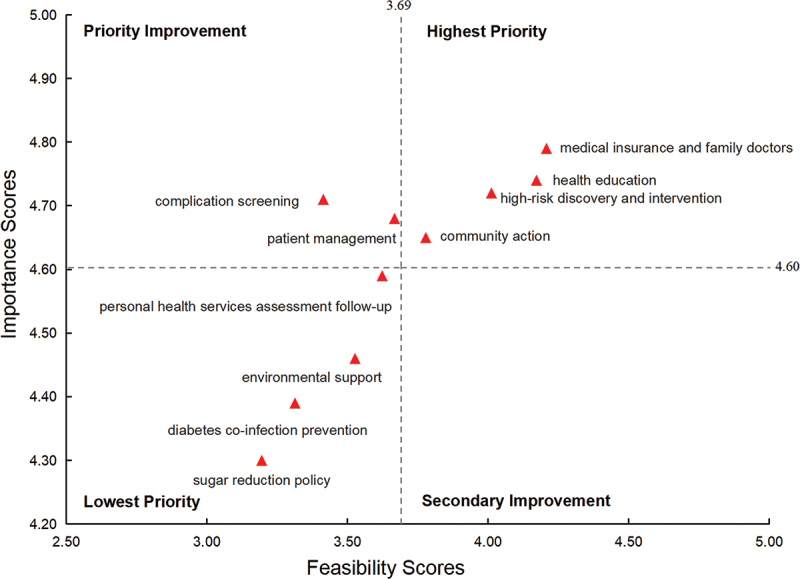
Diabetes prevention and control dimension indicators IPA model.

Descriptive statistics of importance and feasibility scores for all measures are presented in Table 1. The average scores for feasibility and importance were 3.69 and 4.60, respectively. Based on these scores, the matrix was divided into four quadrants, with ten dimensions allocated accordingly (Figure 2).

**Table 1.** Feasibility and importance scores of different dimensions of diabetes prevention and control.

Level and Dimension indicators	Feasibility scores	Importance scores
individual level		
high-risk discovery and intervention	4.01 ± 0.69	4.72 ± 0.45
patient management	3.67 ± 0.80	4.68 ± 0.51
complication screening	3.41 ± 0.98	4.71 ± 0.57
diabetes co-infection prevention	3.31 ± 0.97	4.39 ± 0.79
population level		
community action	3.78 ± 0.93	4.65 ± 0.63
sugar reduction policy	3.20 ± 1.06	4.30 ± 0.84
environmental support	3.53 ± 0.93	4.46 ± 0.69
health education	4.17 ± 0.75	4.74 ± 0.47
personal health services assessment follow-up	3.61 ± 0.96	4.59 ± 0.71
medical insurance and family doctors	4.21 ± 0.71	4.79 ± 0.43

Quadrant 1 (‘Highest Priority’), included dimensions such as medical insurance and family doctors, health education, high-risk discovery and intervention and community action. Quadrant 2 (‘Priority Improvement’), contained patient management and complication screening. Quadrant 3 (‘Lowest Priority’), included dimensions such as personal health services assessment follow-up, environmental support, diabetes co-infection prevention, and sugar reduction policy. Quadrant 4 (‘Secondary Improvement’), contained no dimension indicators in this study.

Measures such as health education, community action, high-risk discovery and intervention, and medical insurance with family doctors demonstrated high scores in both importance and feasibility, supported by strong government policies, well-established public health infrastructure, and integration into primary care. In contrast, patient management and complication screening, while highly important, exhibited lower feasibility due to higher demands for personnel, technical expertise, and continuous monitoring. Other measures, including environmental support, sugar reduction policies, personal health services follow-up, and co-infection prevention, received lower scores in both importance and feasibility, reflecting limited policy attention, insufficient resources, and higher implementation complexity.

### Ranking of different dimension indicators

**Figure 3.**
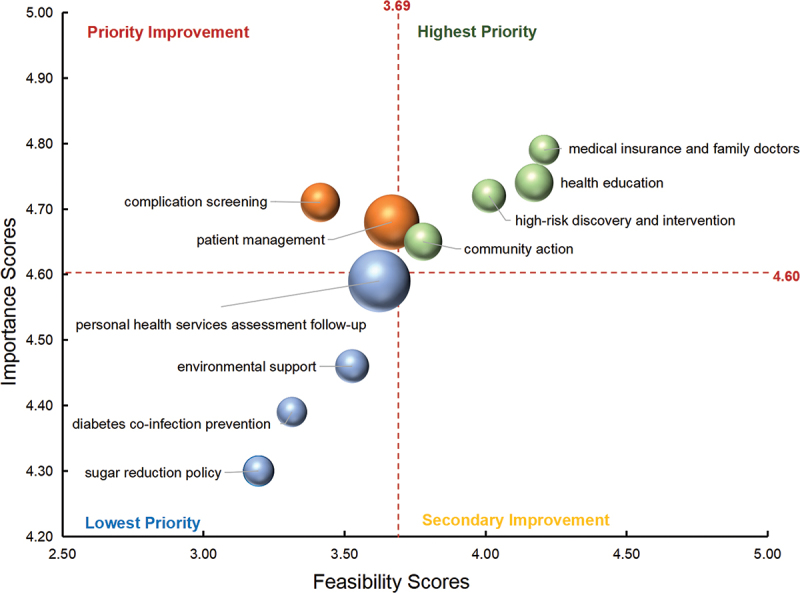
Priority indicators for the diabetes prevention and control dimension bubble chart.

The results of the second-order confirmatory factor analysis model are shown in Supplementary Fig S1. Within Quadrants 1, 2, and 3, the dimensions with the highest standardised factor loadings were health education (0.871), patient management (0.943), and personal health service assessment follow-up (0.967), respectively. These dimensions were considered the highest priority within their respective quadrants and should be given the utmost consideration. Figure 3 shows the specific prioritization order of dimensions within each quadrant.

### Interrelationships of dimension indicators

**Figure 4.**
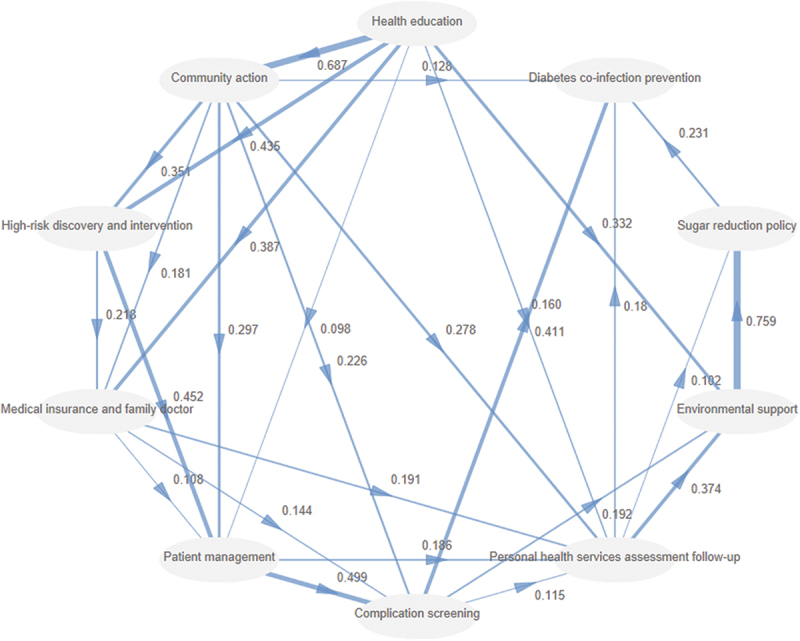
Diabetes prevention and control dimension indicator path diagram.

Based on the prioritisation of the dimensions mentioned above, the results of the PLS-SEM revealed the following relationships: health education positively influenced community action (β = 0.687, *p* < 0.001), high-risk discovery and intervention (β = 0.435, *p* < 0.001), environmental support had a positive effect on sugar reduction policy (β = 0.759, *p* < 0.001), patient management positively influenced complication screening (β = 0.499, *p* < 0.001), high-risk discovery and intervention had a positive effect on patient management (β = 0.452, *p* < 0.001), and complication screening positively influenced diabetes co-infection prevention (β = 0.411, *p* < 0.001). Figure 4 visualizes the path relationships among dimensions derived from the PLS-SEM. Supplementary Table S2 provides the detailed path coefficients.

Further analysis revealed that health education had the following total effect values on other dimension indicators: 0.687 on community action, 0.676 on high-risk discovery and intervention, 0.658 on medical insurance and family doctor, 0.679 on patient management, 0.588 on complication screening, 0.671 on personal health services assessment follow-up, 0.695 on environmental support, 0.596 on sugar reduction policy, and 0.588 on diabetes co-infection prevention. These findings indicated that health education exerts a central and widespread influence across the entire diabetes prevention and control system. Supplementary Figure S2 and Table S3 show the direct, indirect, and total effects between dimensions.

### Sorting of measure indicators

**Figure 5.**
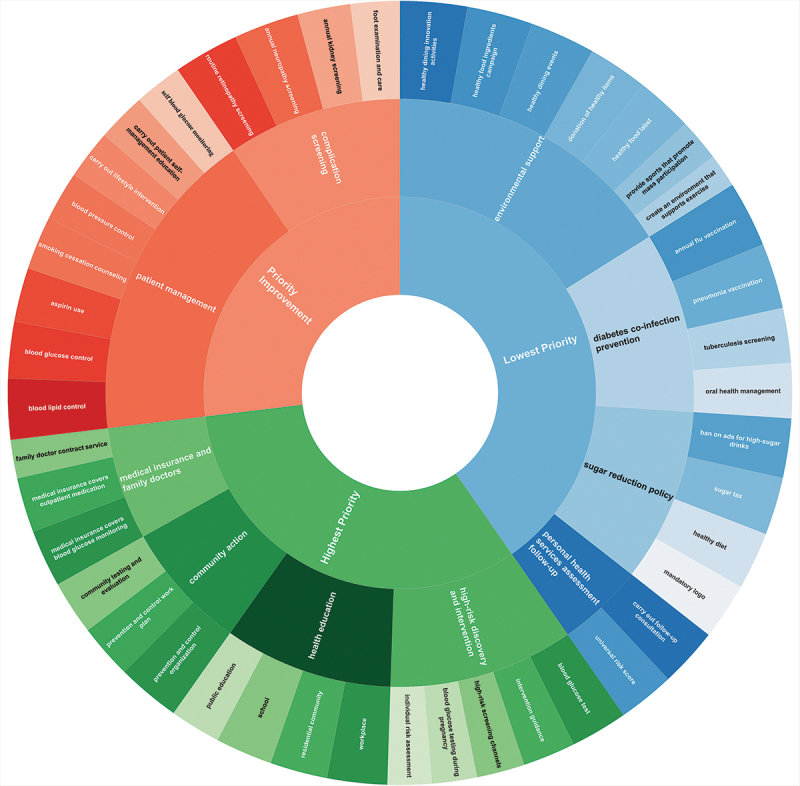
Sunburst chart displaying the prioritization of diabetes prevention and control measures based on feasibility and standardized factor loadings. The innermost ring represents the IPA quadrant classification: green for highest priority, red for priority Improvement, and blue for Lowest priority. The middle ring displays the first-order dimensions within each quadrant, while the outermost ring shows the individual measures under each dimension. Within each layer, the same color depth indicates the relative ranking based on standardized factor loadings: darker shades represent higher priority within the quadrant.

Based on the ranking of standardised first-order factor loadings, the prioritised measures within different quadrants were as follows: (1) ‘Highest Priority’: workplace, prevention and control organization, blood glucose test and medical insurance covers blood glucose monitoring; (2) ‘Priority Improvement’: blood lipid control and routine retinopathy screening; and (3) ‘Lowest Priority’: carry out follow-up consultation, healthy dining innovation activities, ban on ads for high-sugar drinks, and annual influenza vaccination. Figure 5 presents the full prioritization order of indicators within the sunburst chart.

## Discussion

This study aimed to assess and prioritize diabetes prevention and control measures in China, focusing on their feasibility and importance. Using the IPA model and latent variable analysis techniques, we identified key dimensions and measures that should guide future diabetes prevention and control efforts in the China’s pilot demonstration areas. Our findings provide valuable insights into the strategic prioritization of resources and actions in diabetes prevention, especially within large-scale public health initiatives.

The IPA model has been widely used across various fields, including preventive care services [19], health service quality [20], and patient experience [21], to evaluate the relative importance and performance of different factors. The model’s ability to visually categorize variables into quadrants based on their perceived importance and performance makes it a useful tool for prioritizing actions and guiding resource allocation.

To the best of our knowledge, this is the first systematic study to evaluate and prioritize diabetes prevention and control measures. Currently, there are no published studies that specifically assess the feasibility and prioritization of such measures, particularly in the context of large-scale national demonstration areas. Although similar research has not yet been conducted, the findings of other related studies provide additional support for the prioritization of the measures identified in our study. Our results indicate that health education, community action, high-risk discovery and intervention, and medical insurance and family doctors are the highest-priority areas for diabetes prevention and control. These dimensions are characterized by both high importance and high feasibility, which aligns with the global focus on strengthening community-level interventions and improving access to healthcare services. For instance, health education also plays a crucial role by fostering the understanding of the nature of diabetes, treatment strategies, risk factors, and associated complications. This knowledge improves health literacy, empowers individuals to manage their condition, and reduces the occurrence of complications, ultimately lowering the incidence and mortality rates of diabetes [22,23].

A notable positive correlation was observed between importance and feasibility scores across the evaluated dimensions. Although these two constructs are conceptually distinct, several factors may account for their alignment in this study. First, the measures included were rigorously pre-selected through a systematic evidence review and alignment with national priorities, which inherently limited the variance in perceived importance. Second, in China’s centralized health system, interventions recognized as important are often accompanied by substantial resource allocation, policy endorsement, and institutional support, thereby enhancing their perceived feasibility. Third, the simultaneous assessment of both constructs by the same respondents may introduce common method bias, where perceived importance may influence feasibility ratings. Lastly, even within this overall correlation, individual feasibility sub-dimensions revealed specific implementation challenges that deserve targeted attention. Future research should employ independent, longitudinal, and multi-source evaluation designs to better disentangle importance from feasibility in public health intervention assessments.

The CFA model also showed that health education should be prioritized for implementation. The high feasibility of health education observed in this study may reflect several contextual advantages in China. First, China has established a robust public health infrastructure at the community level, including extensive networks of community health service centers, township health centers, and village clinics, which provide ready platforms for delivering health education interventions. Second, health education initiatives have been strongly supported by national policies such as the Healthy China Action (2019–2030), which mandates health promotion and literacy improvement as core priorities. Third, cultural factors may contribute to the acceptance and effectiveness of health education, as collective community engagement and top-down public health campaigns have long-standing social legitimacy in the Chinese health governance system. Furthermore, the availability of mass media, mobile health platforms, and government-sponsored information campaigns has facilitated widespread dissemination of health education materials, further enhancing feasibility. In addition, the community-oriented diabetes prevention programs contribute to the promotion of health practices [24], as evidenced by studies indicating that interventions led by community health workers can improve blood glucose levels in patients [25–27]. Early intervention targeting individuals at high risk of diabetes is imperative to prevent or delay the onset of diabetes and its associated complications [28,29]. Additionally, China’s policy of reimbursing outpatient diabetes medications and the implementation of family doctor services in certain demonstration areas support the feasibility and rationality of our prioritization. By sustaining and expanding these high-priority measures, the healthcare system can help mitigate the long-term burden of diabetes-related complications.

Interestingly, no measures fell into the ‘Secondary Improvement’ quadrant (high importance but low feasibility) in our IPA matrix. This phenomenon may be attributed to several contextual factors. First, the selection of interventions in this study was based on a rigorous, multi-step process involving systematic literature review, expert consensus, and alignment with national guidelines and policies. This approach inherently filtered out interventions that were known to be important but lacked clear paths to implementation, thus reducing the likelihood of identifying measures with high importance yet low feasibility. Second, China’s top-down public health governance structure plays a critical role. Measures identified as important at the national level typically receive substantial policy support, resource investment, and institutional backing, which increases their perceived feasibility among local implementers. This strong alignment between national priorities and local execution capacity may compress the space for ‘important but unfeasible’ strategies to emerge in routine practice. Lastly, the joint assessment of importance and feasibility by the same respondents may introduce response patterns that overestimate feasibility for measures already widely promoted. This potential bias highlights the need for future studies to adopt independent or staggered evaluation methods to more clearly disentangle these constructs.

In the ‘Priority Improvement’ quadrant, which includes patient management and complication screening, emphasizes the importance of refining and enhancing existing interventions in these areas. These two indicators indicate that patient management is more important in CFA. Diabetes self-management, which includes including dietary management, blood glucose test, lifestyle changes, physical activity, and smoking cessation, is a critical factor in controlling the growing prevalence of diabetes [30]. Systematic review has shown that self-management among patients with type 2 diabetes can reduce the overall mortality risk [31]. In 2023, the ‘Standards of Medical Care in Diabetes’ proposed by the American Diabetes Association emphasize more proactive management of lipids and blood pressure, and the use of low-dose aspirin for diabetes patients with cardiovascular disease [32]. These findings underscore the significance of blood lipid and glucose control and aspirin use, in diabetes management. The prevalence of diabetes-related complications is high and irreversible, making screening crucial for the prevention and management of the occurrence and progression of diabetes and its complications [33]. Although these dimensions are considered less feasible than those in the ‘Highest Priority’ quadrant, they remain crucial and require focused efforts to improve their implementation.

On the other hand, dimensions such as personal health services assessment follow-up, environmental support, and diabetes co-infection prevention, which were placed in the ‘Lowest Priority’ quadrant, suggest that these measures are not as feasible or immediately impactful as others. This may reflect their lower immediate relevance to the current needs of diabetes prevention and control in the pilot areas, though they may be more important in long-term strategies. When resources allow for the implementation of additional measures, the CFA model suggests that prioritizing personal health services assessment follow-up should be given special consideration.

The prioritisation of diabetes prevention and control measures, as identified in this study, reveals the interconnectedness of strategies rather than their isolated implementation. The implementation of prioritised measures positively influenced the implementation of other measures. The strong and widespread total effects of health education observed in this study highlight its central role in the diabetes prevention and control system. Not only does health education directly promote community action (β = 0.687), but it also significantly influences high-risk discovery and intervention (total effect = 0.676), patient management (total effect = 0.679), complication screening (total effect = 0.588), medical insurance and family doctors (total effect = 0.658), personal health services follow-up (total effect = 0.671), environmental support (total effect = 0.695), sugar reduction policy (total effect = 0.596), and diabetes co-infection prevention (total effect = 0.588). This suggests that improving health literacy serves as a key leverage point capable of driving improvements across multiple layers of the prevention system. Similar cascading effects of health education have been reported in global non-communicable disease prevention programs [34], emphasizing the importance of embedding educational interventions as a foundation for comprehensive diabetes control policies. These findings underscore the need for mutual reinforcement among measures, highlighting the need for a comprehensive approach to enhancing the effectiveness of diabetes prevention and control.

This study demonstrates a feasible approach to prioritizing diabetes prevention and control measures by assessing both their importance and feasibility at the implementation level. For low- and middle-income countries (LMICs), where resources are constrained, this two-dimensional prioritization can help decision-makers focus on interventions that are not only impactful but also practical to implement. China’s experience in aligning national strategies with community-level capacity, and in emphasizing health education, community engagement, and early risk detection, may serve as a reference for LMICs seeking scalable, evidence-informed chronic disease control strategies.

Strengths of this study include the following three areas: (1) It is the first time to evaluate diabetes prevention and control measures from a feasibility perspective facilitates the efficient utilisation of limited resources and maximises the benefits of prevention and control efforts. (2) The assessment of control measures by core professionals in chronic disease prevention and control within demonstration areas who bring their expertise and consider local contextual factors enhances the practical implementation of the measures. (3) The novel application of latent variable analysis techniques such as confirmatory factor analysis and path analysis for prioritising control measures provides innovative approaches for priority ranking within the healthcare system.

It is imperative to acknowledge that this study was based on the local context of China’s demonstration areas, which introduces certain limitations. First, the application of the findings requires consideration of various factors, including local policies and relevant circumstances. Second, the feasibility assessments relied on subjective judgments from the participating professionals, which may have introduced potential bias. As public health professionals, their assessments may have been influenced by organizational priorities or alignment with national policy goals, rather than solely by implementation experience. Although this bias cannot be fully eliminated, the anonymous survey design and broad national sample help reduce its potential impact. Additionally, the systematic online survey format prevented us from gathering demographic information about the respondents, limiting the ability to analyze the data from a demographic perspective. Third, our study focused on the feasibility and importance of different measures, but we did not explore the specific barriers and challenges faced during their implementation. Future research should address these barriers to provide deeper insights into improving the practical application of these measures. To address these limitations, we plan to collect other data (such as economy) from different provinces and further analyse them in conjunction with the execution of practical measures.

## Conclusions

The feasibility evaluation of diabetes prevention and control measures in China’s demonstration areas conducted in this study is beneficial for improving their efficiency and facilitating the implementation of feasible measures. The use of the IPA model enables the prioritisation of diabetes prevention and control measures and combined with confirmatory factor analysis, it allows for a more comprehensive ranking. The innovative approach to priority ranking presented in this study has significant implications for healthcare system management. This study offers valuable insights for policymakers and health professionals involved in diabetes prevention, helping to ensure that interventions are both effective and feasible. Future research should aim to build on these findings by conducting longitudinal studies to track the implementation and outcomes of prioritized measures over time. Such studies would provide more robust evidence on the actual effectiveness, sustainability, and real-world impact of the interventions, thereby informing continuous refinement and scaling-up of diabetes control strategies.

## Supplementary Material

Supplemental Material

## Data Availability

The datasets used and/or analysed during the current study are available from the corresponding author on reasonable request.
