# Supplementary material for: Feasibility and priority strategies for diabetes prevention and control in Chinese demonstration areas
Source: Glob Health Action. 2025 Aug 20;18(1):2543605. doi: 10.1080/16549716.2025.2543605 (PMC12372481; doi:10.1080/16549716.2025.2543605)
Supplement: Supplemental Material [file ZGHA_A_2543605_SM5570.docx]

**Feasibility and priority strategies for diabetes prevention and control in Chinese demonstration areas**

**Supplemental Material**

Jing Li, Jing Yang, Jiayu Feng, Xiaohui Xu, Tingling Xu, Maigeng Zhou, Wenlan Dong*, Yanbo Zhang*

Table S1 Database Survey of Diabetes Prevention and Control Measures

| **No.** | **Category of measures** | **Specific measures description** | **Importance** | **Feasibility** | | | |
| --- | --- | --- | --- | --- | --- | --- | --- |
|  |  |  |  | **Accessibility** | **Technical simplicity** | **Economic feasibility** | **Cultural acceptability** |
| 1 | **Individual levels** | | | | | | |
| 1.1 | **high-risk discovery and intervention** | | | | | | |
| 1.1.1 | **high-risk screening channels** | 1. Identify high-risk individuals for diabetes through channels such as health records, basic public health services, and opportunistic screening (including health check-ups and routine medical consultations). |  |  |  |  |  |
| 1.1.2 | **individual risk assessment** | 1. Use the China Diabetes Risk Score (CDRS) to assess the risk of diabetes in the general population aged 20-74 years. The score ranges from 0 to 51, and individuals with a score ≥ 25 should undergo an Oral Glucose Tolerance Test (OGTT). |  |  |  |  |  |
| 1.1.3 | **blood glucose test** | 1. Conduct blood glucose tests for high-risk individuals and provide lifestyle advice. |  |  |  |  |  |
| 1.1.4 | **blood glucose testing during pregnancy** | 1. Perform blood glucose screening at the first prenatal check-up or between 24-28 weeks of gestation, and offer lifestyle advice or metformin recommendations. |  |  |  |  |  |
| 1.2 | **patient management** | | | | | | |
| 1.2.1 | **intervention guidance** | 1. Implement systematic lifestyle interventions, including consultations and guidance on diet, exercise, and weight management provided by healthcare professionals, with regular blood glucose monitoring. |  |  |  |  |  |
| 1.2.2 | **carry out lifestyle intervention** | 1. Strengthen lifestyle interventions and maintain an ideal body weight; provide medical nutrition therapy (personalized nutrition assessment, diagnosis, and intervention); offer exercise prescriptions (personalized exercise assessment and intervention). |  |  |  |  |  |
| 1.2.3 | **carry out patient self-management education** | 1. Through individual education, group education, a combination of individual and group education, as well as remote education, patients are empowered to fully understand diabetes and acquire self-management skills, thereby enhancing their self-efficacy. |  |  |  |  |  |
| 1.2.4 | **self blood glucose monitoring** | 1. Encourage patients to monitor their blood glucose at home to assess blood glucose control and fluctuations, providing personalized treatment and facilitating necessary adjustments to achieve target glucose levels. |  |  |  |  |  |
| 1.2.5 | **blood glucose control** | 1. Maintain HbA1c levels below 7.0% or an individualized target. |  |  |  |  |  |
| 1.2.6 | **blood pressure control** | 1. Maintain blood pressure below 140/80 mmHg or an individualized target. |  |  |  |  |  |
| 1.2.7 | **blood lipid control** | 1. Set personalized targets for each patient based on individual needs and conditions. |  |  |  |  |  |
| 1.2.8 | **aspirin use** | 1. Recommend the use of medications where appropriate based on clinical indications. |  |  |  |  |  |
| 1.2.9 | **smoking cessation counseling** | 1. Implement the 5As (Ask, Advise, Assess, Assist, Arrange follow-up) and 5Rs (Relevance, Risk, Reward, Roadblocks, Repeat) in patient management. |  |  |  |  |  |
| 1.3 | **complication screening** | | | | | | |
| 1.3.1 | **routine retinopathy screening** | 1. Type 2 diabetes patients should undergo an initial comprehensive eye examination at diagnosis. Those without diabetic retinopathy should have follow-up examinations every 1-2 years, while those with retinopathy should undergo more frequent monitoring. |  |  |  |  |  |
| 1.3.2 | **annual neuropathy screening** | 1. Type 2 diabetes patients should be screened for diabetic neuropathy at diagnosis, and Type 1 diabetes patients should be screened 5 years after diagnosis. Subsequent screenings should occur at least annually. |  |  |  |  |  |
| 1.3.3 | **foot examination and care** | 1. Conduct a comprehensive annual foot examination for all diabetes patients, including a detailed inquiry into the history of macrovascular and microvascular complications. Assess current symptoms of neuropathy (such as pain, burning, and numbness) and peripheral vascular disease (such as lower limb fatigue and claudication) to identify risk factors for ulcers and amputations. |  |  |  |  |  |
| 1.3.4 | **annual kidney screening** | 1. After a diagnosis of Type 2 diabetes, patients should undergo annual kidney disease screening, including routine urine tests, urine albumin-to-creatinine ratio (UACR), and blood creatinine (with calculation of estimated glomerular filtration rate, eGFR). |  |  |  |  |  |
| 1.4 | **diabetes co-infection prevention** | | | | | | |
| 1.4.1 | **annual flu vaccination** | 1. All diabetic patients aged 6 months and older should receive an annual influenza vaccination. |  |  |  |  |  |
| 1.4.2 | **pneumonia vaccination** | 1. Diabetic patients aged 2 years and older should receive pneumococcal polysaccharide vaccination. Patients aged 65 or older who were previously vaccinated more than 5 years ago should receive a booster dose. |  |  |  |  |  |
| 1.4.3 | **tuberculosis screening** | 1. The WHO recommends tuberculosis screening for patients with diabetes. |  |  |  |  |  |
| 1.4.4 | **oral health management** | 1. Pay attention to the oral health of diabetic patients. |  |  |  |  |  |
| 2 | **Population level** | | | | | | |
| 2.1 | **community action** | | | | | | |
| 2.1.1 | **prevention and control organization** | 1. Establish a diabetes prevention and control working group, or a broader chronic disease prevention group, involving relevant government departments, healthcare institutions, and social organizations. |  |  |  |  |  |
| 2.1.2 | **prevention and control work plan** | 1. Formulate diabetes prevention and control plans, or more comprehensive chronic disease prevention plans that include diabetes. |  |  |  |  |  |
| 2.1.3 | **community monitoring and evaluation** | 1. Continuously collect and analyze data on the prevalence of diabetes and associated risk factors in the community to identify health issues related to diabetes and provide evidence for decision-making. |  |  |  |  |  |
| 2.2 | **sugar reduction policy** | | | | | | |
| 2.2.1 | **sugar tax** | 1. Impose taxes on sugar-sweetened beverages |  |  |  |  |  |
| 2.2.2 | **ban on ads for high-sugar drinks** | 1. Ban advertising of high-sugar beverages (e.g., sugary sodas, fruit juices, flavored yogurts, instant coffee) through media channels such as radio, print, outdoor, and online platforms. |  |  |  |  |  |
| 2.2.3 | **mandatory logo** | 1. Include mandatory labeling for sugar content (e.g., sucrose) on pre-packaged food products. |  |  |  |  |  |
| 2.2.4 | **healthy diet** | 1. Implement policies promoting healthy diets, such as encouraging the consumption of whole grains over refined starches and reducing the intake of sugary drinks, saturated fats, and high-calorie foods. Government events should adhere to healthy dietary guidelines. |  |  |  |  |  |
| 2.3 | **environmental support** | | | | | | |
| 2.3.1 | **healthy food label** | 1. Introduce prominent labeling on food packaging to indicate whether a product is healthy, making it easier for consumers to make healthier choices. |  |  |  |  |  |
| 2.3.2 | **healthy dining events** | 1. Encourage food service companies to offer healthier meal options to consumers, including low-calorie, low-sugar meals made with whole grains and healthier cooking oils. Companies participating in this initiative can apply for corresponding subsidies. |  |  |  |  |  |
| 2.3.3 | **healthy food ingredients campaign** | 1. Support food companies in innovating and developing healthier products that cater to local tastes. Participating companies may apply for corresponding subsidies. |  |  |  |  |  |
| 2.3.4 | **healthy dining innovation activities** | 1. Support restaurants in developing and promoting healthier menus under the guidance of healthy eating programs. Participating companies may apply for corresponding subsidies. |  |  |  |  |  |
| 2.3.5 | **donation of healthy items** | 1. Guide charitable organizations, welfare groups, community organizations, and individual donors to select healthier food options for donations. |  |  |  |  |  |
| 2.3.6 | **create an environment that supports exercise** | 1. Provide free community spaces for physical activity (e.g., parks) to encourage residents to engage in regular physical exercise. |  |  |  |  |  |
| 2.3.7 | **provide sports that promote mass participation** | 1. Organize walking challenges to encourage more community and individual participation. |  |  |  |  |  |
| 2.4 | **health education** | | | | | | |
| 2.4.1 | **public education** | 1. Use both new media and traditional media to launch public health education campaigns aimed at diabetes prevention. |  |  |  |  |  |
| 2.4.2 | **school** | 1. Offer diabetes prevention courses in schools and for adolescents at an early stage. |  |  |  |  |  |
| 2.4.3 | **workplace** | 1. Implement educational interventions on diabetes-related risk factors for the working population after employment. |  |  |  |  |  |
| 2.4.4 | **residential community** | 1. Provide self-management guidelines and education to patients through community health service centers. |  |  |  |  |  |
| 2.5 | **personal health services assessment follow-up** | | | | | | |
| 2.5.1 | **universal risk score** | 1. Promote the use of the China Diabetes Risk Score for assessing the risk of diabetes in the general population. |  |  |  |  |  |
| 2.5.2 | **carry out follow-up consultation** | 1. Implement enhanced screening for high-risk individuals, with free or low-cost follow-up consultations after initial screening. |  |  |  |  |  |
| 2.6 | **medical insurance and family doctors** | | | | | | |
| 2.6.1 | **medical insurance covers blood glucose monitoring** | 1. Include the cost of self-monitoring blood glucose strips and lancets in insurance coverage for diabetes patients. |  |  |  |  |  |
| 2.6.2 | **medical insurance covers outpatient medication** | 1. The health insurance coverage includes outpatient medications for diabetes patients. |  |  |  |  |  |
| 2.6.3 | **family doctor contract service** | 1. Provide annual health check-ups for patients with diagnosed Type 2 diabetes through family doctor services, including four free fasting blood glucose tests and at least four face-to-face follow-up visits annually. |  |  |  |  |  |


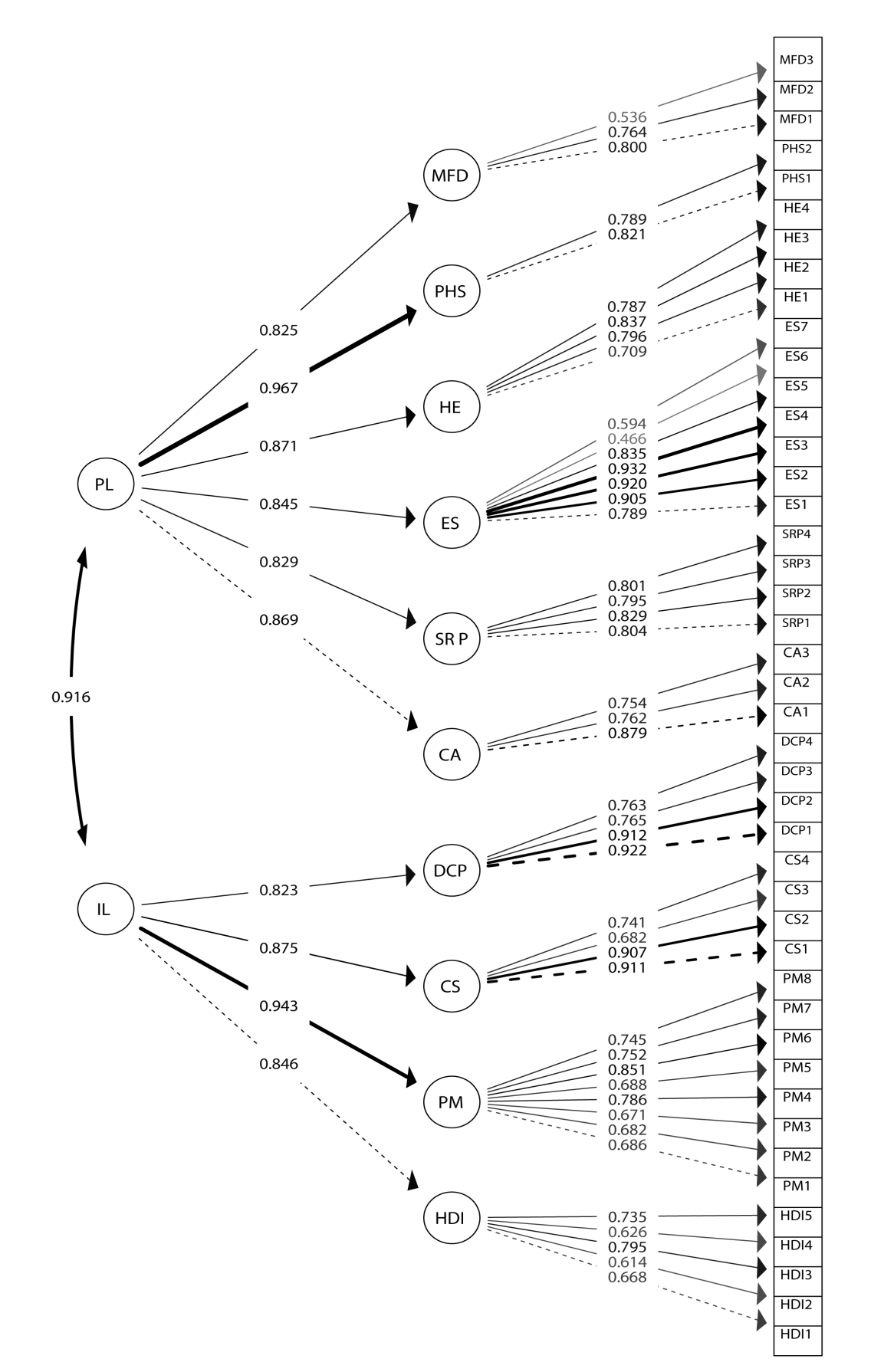


Fig S1 Confirmatory factor analysis model of diabetes prevention and control measures

PL, Population level; IL, Individual level; HDI, high-risk discovery and intervention; PM, patient management; CS, complication screening; DCP, diabetes co-infection prevention; CA, community action; SRP, sugar reduction policy; ES, environmental support; HE, health education; PHS, personal health services assessment follow-up; MFD, medical insurance and family doctors; HDI1, high-risk screening channels; HDI2, individual risk assessment; HDI3, blood glucose test; HDI4, blood glucose testing during pregnancy; HDI5, intervention guidance; PM1, carry out lifestyle intervention; PM2, carry out patient self-management education; PM3, self blood glucose monitoring; PM4, blood glucose control; PM5, blood pressure control; PM6, blood lipid control; PM7, aspirin use; PM8, smoking cessation counseling; CA1, routine retinopathy screening; CA2, annual neuropathy screening; CA3, foot examination and care; CA4, annual kidney screening; DCP1, annual flu vaccination; DCP2, tuberculosis screening; DCP3, pneumonia vaccination;DCP4, oral health management; CA1, prevention and control organization; CA2, prevention and control work plan; CA3, community testing and evaluation; SRP1,sugar tax; SRP2, ban on ads for high-sugar drinks; SRP3, mandatory logo; SRP4, healthy diet; ES1, healthy food label; ES2, healthy dining events; ES3, healthy food ingredients campaign; ES4, healthy dining innovation activities; ES5, donation of healthy items; ES6, create an environment that supports exercise; ES7, provide sports that promote mass participation; HE1, public education; HE2, school; HE3, workplace; HE4, residential community;PHS1, universal risk score; PHS2, carry out follow-up consultation; MFD1, medical insurance covers blood glucose monitoring; MFD2, medical insurance covers outpatient medication; MFD3, family doctor contract service


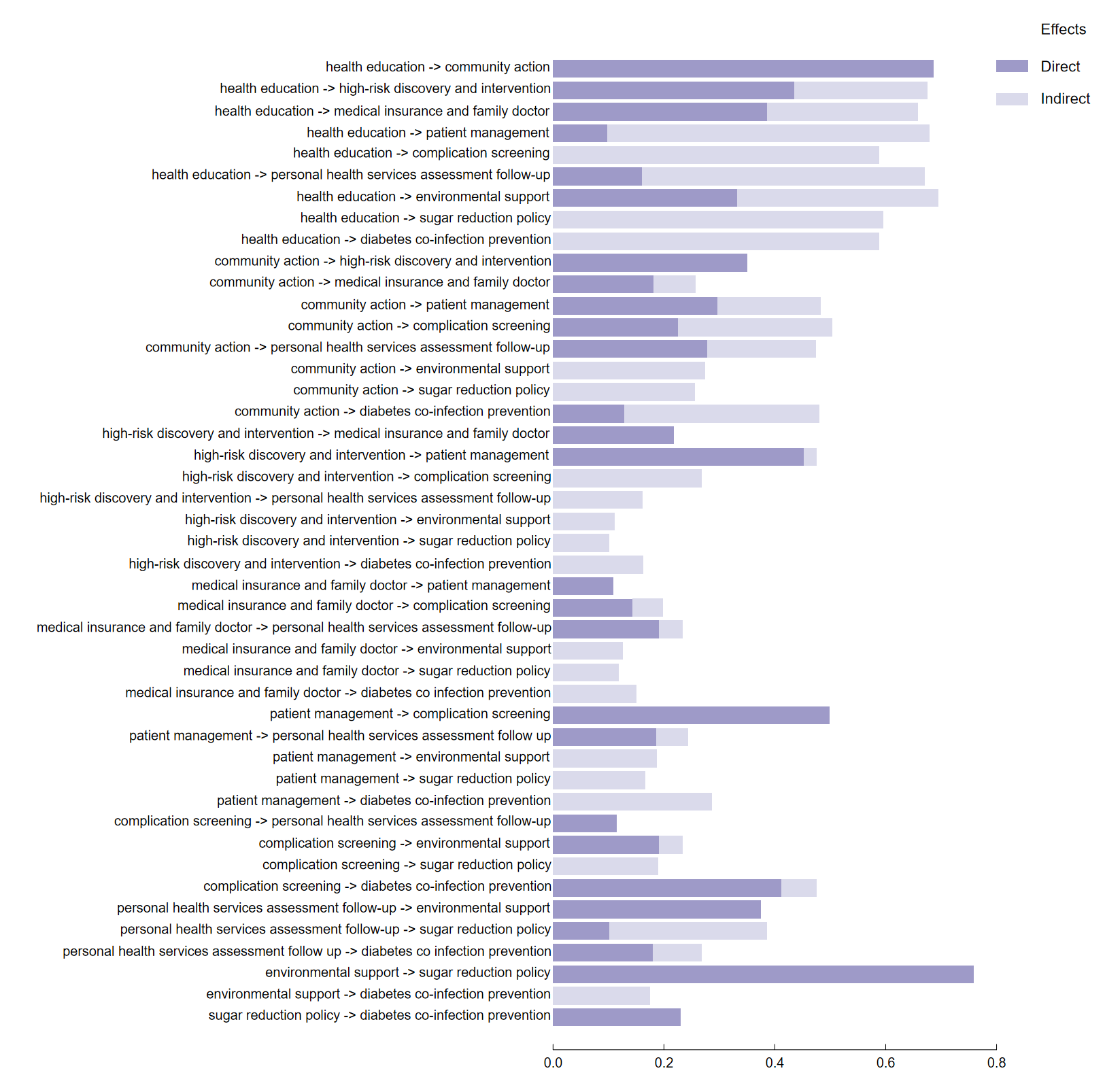


Fig S2 The direct effect, indirect effect and total effect of dimension indicators

Table S2 Estimations of PLS-SEM path coefficients between dimension indicators

| Path | $\beta$ | Std | $t$ | *P* |
| --- | --- | --- | --- | --- |
| health education->community action | 0.687 | 0.034 | 20.439 | <0.001 |
| health education->high-risk discovery and intervention | 0.435 | 0.044 | 9.855 | <0.001 |
| health education->medical insurance and family doctor | 0.387 | 0.050 | 7.715 | <0.001 |
| health education->patient management | 0.098 | 0.041 | 2.401 | 0.017 |
| health education->personal health services assessment follow-up | 0.160 | 0.044 | 3.596 | <0.001 |
| health education->environmental support | 0.332 | 0.040 | 8.221 | <0.001 |
| community action->high-risk discovery and intervention | 0.351 | 0.044 | 7.958 | <0.001 |
| community action->medical insurance and family doctor | 0.181 | 0.049 | 3.718 | <0.001 |
| community action->patient management | 0.297 | 0.038 | 7.822 | <0.001 |
| community action->complication screening | 0.226 | 0.043 | 5.221 | <0.001 |
| community action->personal health services assessment follow-up | 0.278 | 0.045 | 6.108 | <0.001 |
| community action->diabetes co-infection prevention | 0.128 | 0.041 | 3.105 | 0.002 |
| high-risk discovery and intervention->medical insurance and family doctor | 0.218 | 0.048 | 4.546 | <0.001 |
| high-risk discovery and intervention->patient management | 0.452 | 0.038 | 12.008 | <0.001 |
| medical insurance and family doctor->patient management | 0.108 | 0.036 | 3.035 | 0.003 |
| medical insurance and family doctor->complication screening | 0.144 | 0.038 | 3.768 | <0.001 |
| medical insurance and family doctor->personal health services assessment follow-up | 0.191 | 0.040 | 4.784 | <0.001 |
| patient management->complication screening | 0.499 | 0.045 | 11.174 | <0.001 |
| patient management->personal health services assessment follow-up | 0.187 | 0.050 | 3.742 | <0.001 |
| complication screening->personal health services assessment follow-up | 0.115 | 0.045 | 2.543 | 0.011 |
| complication screening->environmental support | 0.192 | 0.039 | 4.879 | <0.001 |
| complication screening->diabetes co-infection prevention | 0.411 | 0.039 | 10.504 | <0.001 |
| personal health services assessment follow-up->environmental support | 0.375 | 0.042 | 8.900 | <0.001 |
| personal health services assessment follow-up->sugar reduction policy | 0.102 | 0.037 | 2.761 | 0.006 |
| personal health services assessment follow-up->diabetes co-infection prevention | 0.180 | 0.043 | 4.176 | <0.001 |
| environmental support->sugar reduction policy | 0.759 | 0.037 | 20.631 | <0.001 |
| sugar reduction policy->diabetes co-infection prevention | 0.231 | 0.037 | 6.271 | <0.001 |

Table S3 PLS-SEM path direct effect, indirect effect, total effect estimations of dimension indicators

| Path | Direct | Indirect | Total |
| --- | --- | --- | --- |
| health education -> community action | 0.687 | 0.000 | 0.687 |
| health education -> high-risk discovery and intervention | 0.431 | 0.241 | 0.676 |
| health education -> medical insurance and family doctor | 0.387 | 0.271 | 0.658 |
| health education -> patient management | 0.099 | 0.581 | 0.679 |
| health education -> complication screening | 0.053 | 0.588 | 0.588 |
| health education -> personal health services assessment follow-up | 0.149 | 0.511 | 0.671 |
| health education -> environmental support | 0.302 | 0.364 | 0.695 |
| health education -> sugar reduction policy | 0.000 | 0.596 | 0.596 |
| health education -> diabetes co-infection prevention | 0.000 | 0.588 | 0.588 |
| community action -> high-risk discovery and intervention | 0.356 | 0.000 | 0.351 |
| community action -> medical insurance and family doctor | 0.183 | 0.076 | 0.257 |
| community action -> patient management | 0.293 | 0.187 | 0.483 |
| community action -> complication screening | 0.208 | 0.278 | 0.504 |
| community action -> personal health services assessment follow-up | 0.272 | 0.197 | 0.475 |
| community action -> environmental support | 0.074 | 0.274 | 0.274 |
| community action -> sugar reduction policy | 0.000 | 0.256 | 0.256 |
| community action -> diabetes co-infection prevention | 0.129 | 0.352 | 0.480 |
| high-risk discovery and intervention -> medical insurance and family doctor | 0.214 | 0.000 | 0.218 |
| high-risk discovery and intervention -> patient management | 0.454 | 0.024 | 0.476 |
| high-risk discovery and intervention -> complication screening | 0.000 | 0.269 | 0.268 |
| high-risk discovery and intervention -> personal health services assessment follow-up | 0.053 | 0.161 | 0.161 |
| high-risk discovery and intervention -> environmental support | 0.000 | 0.112 | 0.112 |
| high-risk discovery and intervention -> sugar reduction policy | 0.000 | 0.101 | 0.101 |
| high-risk discovery and intervention -> diabetes co-infection prevention | 0.000 | 0.163 | 0.163 |
| medical insurance and family doctor -> patient management | 0.110 | 0.000 | 0.108 |
| medical insurance and family doctor -> complication screening | 0.129 | 0.054 | 0.198 |
| medical insurance and family doctor -> personal health services assessment follow-up | 0.189 | 0.043 | 0.234 |
| medical insurance and family doctor -> environmental support | 0.040 | 0.126 | 0.126 |
| medical insurance and family doctor -> sugar reduction policy | 0.000 | 0.119 | 0.119 |
| medical insurance and family doctor -> diabetes co-infection prevention | 0.000 | 0.151 | 0.151 |
| patient management -> complication screening | 0.484 | 0.000 | 0.499 |
| patient management -> personal health services assessment follow-up | 0.158 | 0.057 | 0.244 |
| patient management -> environmental support | 0.000 | 0.187 | 0.187 |
| patient management -> sugar reduction policy | 0.000 | 0.167 | 0.167 |
| patient management -> diabetes co-infection prevention | 0.000 | 0.287 | 0.287 |
| complication screening -> personal health services assessment follow-up | 0.115 | 0.000 | 0.115 |
| complication screening -> environmental support | 0.163 | 0.043 | 0.234 |
| complication screening -> sugar reduction policy | 0.000 | 0.190 | 0.190 |
| complication screening -> diabetes co-infection prevention | 0.411 | 0.064 | 0.476 |
| personal health services assessment follow-up -> environmental support | 0.334 | 0.000 | 0.375 |
| personal health services assessment follow-up -> sugar reduction policy | 0.105 | 0.284 | 0.386 |
| personal health services assessment follow-up -> diabetes co-infection prevention | 0.180 | 0.089 | 0.269 |
| environmental support -> sugar reduction policy | 0.755 | 0.000 | 0.759 |
| environmental support -> diabetes co-infection prevention | 0.000 | 0.175 | 0.175 |
| sugar reduction policy -> diabetes co-infection prevention | 0.231 | 0.000 | 0.231 |
